# Prevalence of cardiovascular risk factors and socioeconomic level among public-sector workers in Angola

**DOI:** 10.1186/1471-2458-13-732

**Published:** 2013-08-07

**Authors:** Daniel P Capingana, Pedro Magalhães, Amílcar BT Silva, Mauer AA Gonçalves, Marcelo P Baldo, Sérgio L Rodrigues, Cristóvão CF Simões, Albano VL Ferreira, José G Mill

**Affiliations:** 1 Department of Physiological Sciences, Federal University of Espírito Santo, Espírito Santo, Brazil; 2 Department of Physiological Sciences, Medical School of the Agostinho Neto University, Luanda, Angola

**Keywords:** Cardiovascular risk factors, Socioeconomic status, Angola

## Abstract

**Background:**

Cardiovascular diseases are the leading cause of death in the majority of developed and developing countries. African countries are currently facing an increase in both cardiovascular and transmitted diseases. In addition, cardiovascular risk varies among different socioeconomic groups. Thus, we determined the prevalence of modifiable cardiovascular risk factors in apparently healthy public-sector workers and investigated possible relationships with socioeconomic status.

**Methods:**

We employed a cross-sectional study comprising 42.2% (n = 615) of the public-sector workers at Agostinho Neto University, 48% (n = 294) male and 52% (n= 321) female, with ages between 20 and 72 years and from various socioeconomic groups. The study was conducted from February 2009 to December 2010. Personal, anthropometric, biochemical, hemodynamic, socioeconomic, and physical activity data were collected.

**Results:**

The prevalence rates of cardiovascular risk factors were as follows: hypertension, 45.2% (men 46.3%, women 44.2%, P > 0.05); hypercholesterolemia, 11.1% (men 10.5%, women 11.5%, P > 0.05); low high-density lipoprotein (HDL) cholesterol, 50.1% (men 36.9%, women 62.3%; P < 0.05); hypertriglyceridemia, 10.6% (men 12.6%, women 8.7%, P > 0.05); smoking, 7.2% (men 10.2%, women 4.4%; P < 0.05); diabetes, 5.7% (men 5.5%, women 5.9%, P > 0.05); overweight, 29.3% (men 27.3%, women 31.2%, P > 0.05); obesity, 19.6% (men 9.2%, women 29.0%; P < 0.05); sedentary lifestyle, 87.2% (men 83.0%, women 91,0%, P < 0.05); and left ventricular hypertrophy, 20% (men 32.0%, women 9.0%; P < 0.05). At least one risk factor was present in 27.7% of the sample; 15.2% had two risk factors, and 31.4% had three or more risk factors. Among the individuals with low socioeconomic status, 41.0% had three or more risk factors.

**Conclusions:**

The results of this study suggest the existence of a high prevalence of multiple risk factors for cardiovascular disease in apparently healthy public-sector workers in Angola. The workers in lower socioeconomic groups had higher incidences of hypertension, smoking, and left ventricular hypertrophy.

## Background

Cardiovascular diseases (CVD) are the leading cause of death in the majority of developed and developing countries [1]. A century ago, CVD were responsible for less than 10% of all deaths, whereas today, it accounts for approximately 30% of deaths worldwide, including nearly 40% in high-income countries and approximately 28% in low-and middle-income countries. The global trend in deaths from CVD predicts an estimated rate of 32% for the year 2020, with a greater contribution from middle-and low-income countries compared with high-income countries [2].

The profile of CVD varies widely by country and region, and the age-adjusted mortality rates from are simultaneously declining in high-income countries and increasing in low-and middle-income countries [3]. African countries are facing a rapid growth of CVD and have very limited resources for the creation of public health infrastructures similar to those existing in high-income countries. In addition, there are numerous national priorities that compete with the provision of this type of care, including the stimulation of economic growth, social and political changes, the devastation brought on by diseases such as HIV/AIDS in Sub-Saharan Africa [4], and inside conflicts of political and ethnic origin in several countries.

Socioeconomic status is a complex variable influenced by education, work experience, and financial performance. Nevertheless, clear variations in CVD risk have been reported among different socioeconomic groups [5,6]. The socioeconomic imbalances are reflected in other modifiable factors, such as overweight, obesity, and sedentary lifestyle, which are more prevalent in African-Americans than in other ethnic groups [7]. These conditions are linked to numerous comorbidities that affect CVD risk, such as dyslipidemia, hypertension, diabetes mellitus, and metabolic syndrome [8].

Angola is a country with a life expectancy of 50.3 years [9] and an infant mortality rate of 193.5 per 1,000 children under 5 years of age [10]. In addition to infectious and parasitic diseases, the residents continue to suffer the consequences of the civil war that lasted 41 years and resulted in destruction of the health care and education infrastructure, forced the relocation of people to safer places, and caused the disintegration of many families. All these factors may ultimately contribute to increase the CVD burden. Due to the limited information on CVD in Angola, we decided to determine the prevalence and severity of modifiable cardiovascular risk factors in a sample of apparently healthy public-sector workers at Agostinho Neto University (UAN) in Luanda, Angola, and to investigate the relationships of these factors with socioeconomic status of participants.

## Methods

This study was a cross-sectional, descriptive study of a sample of public-sector working at UAN in Luanda, Angola. The selection of this sample of public-sector workers should facilitate study follow-up because this population is relatively stable.

The research was conducted in accordance with national and international ethical guidelines and approved by the Independent Ethics Committee of the School of Medicine of UAN.

UAN is a public institution of higher education employing 1,458 domestic public-sector workers, of which 49.7% (n = 724) are teachers (514 males and 210 females) and 50.3% (n = 734) are staff (359 males and 375 females), with ages between 20 and 72 years. All workers were invited to participate, and our goal was to include 50% (n = 729) of the eligible population. However, due to difficulties to enroll participants living far from the data collect centre, the study enrolled 42.2% (n = 615) of the eligible population (48% men and 52% women). The sampling method was developed in two stages. The first stage consisted of sending an invitation letter from the Dean of UAN to the chairs of the Schools and Institutes of UAN to promote the study among all public-sector workers and invite them to participate in the study. The second phase consisted of publicizing the study via pamphlets, posters, and meetings between the research team and groups of workers in various public educational institutions. The participation of each public-sector worker in the project was voluntary and was preceded by the reading and signing of an informed consent form. The study was conducted at the Department of Physiological Sciences Teaching and Research at the Medical School of UAN. Three employees from the abovementioned department were trained to obtain all data, including standard measurements of blood pressure, anthropometric parameters, acquisition of electrocardiogram recordings, and assisting of the respondents in correctly completing the questionnaire. Training and certification of staff investigators was conducted by an experienced researcher over five days.

Data were collected from February 2009 to December 2010 based on a modified questionnaire used in the WHO-MONICA Project and on the WHO manuals for stepwise approaches and the surveillance of non-transmitted diseases [11].

Personal, anthropometric, biochemical, hemodynamic, socioeconomic, and physical activity data were collected. The socioeconomic status was classified into quartiles according to average monthly household income [12]: 1st quartile (low socioeconomic class), 2nd quartile (middle class), 3rd quartile (upper middle class), and 4th quartile (upper class). Education was classified into three levels based on the number of years of education: low (≤ 4 years of education), middle (5–12 years of education), and high (≥ 13 years of education).

Individuals who reported use of tobacco (cigarette, cigar, or pipe) on a regular (daily smoking) or occasional basis and those who reported having stopped smoking 6 months prior to the day of the interview were considered as smokers. Individuals who did not report practicing sports for competition or engaging in physical activity for leisure lasting at least 30 minutes at a frequency of three days per week were considered sedentary [13,14].

Systolic blood pressure (SBP) and diastolic blood pressure (DBP) were measured in the left arm by oscillometry three times in each individual, with a minimum interval between measurements of 10 minutes, using an OMRON (model HEM- 705CP,Tokyo, Japan) blood pressure monitor. SBP and DBP levels were calculated as the arithmetic mean of the last two measurements. The VII Joint National Committee criteria were used for BP classification [15]. Thus, individuals with SBP ≥140 mmHg and/or with DBP ≥90 mmHg were classified as hypertensive, in addition to the individuals taking antihypertensive medication, regardless of the frequency of use.

The body weight (W) of each participant was measured in kilograms (kg) using a previously calibrated mechanical scale (SECA) with a maximum capacity of 220 kg and accuracy of 100 grams. The height (H) of each participant was measured in centimeters (cm) using a stadiometer fixed to the SECA scale with an accuracy of 0.5 cm. The waist circumference (WC) and hip circumference (HC) were each measured twice with an inextensible, inelastic, 1-cm-wide tape measure. The following cutoff points were used for body mass index (BMI = W/H^2^) [16]: low body weight, BMI < 18.5 kg/m^2^; normal weight, BMI 18.5 to 24.9 kg/m^2^; overweight, BMI 25 to 29.9 kg/m^2^; and obese, BMI 30 kg/m^2^.

Blood was collected after 10 to 12 hours fasting by deep vessel venipuncture. The plasma glucose level and the lipid profile were determined to obtain the total cholesterol, high-density lipoprotein cholesterol fraction (HDL-c), and triglyceride (TG) levels. The low-density lipoprotein cholesterol fraction (LDL-c) was calculated using the Friedewald equation [17] to triglycerides <400 mg/dL. The cutoff points used to define hypercholesterolemia stages, the reduction of the HDL-c fraction, and hypertriglyceridemia were those recommended by the Third Expert Panel on the Detection, Evaluation, and Treatment of High Blood Cholesterol in Adults [18]; namely, total cholesterol (TC) was considered high for values ≥240 mg/dL, and the HDL-c fraction was considered low for values <40 mg/dL in men and <50 mg/dL in women. In addition, the LDL-c fraction was considered high for values >160 mg/dL.

The TG level was considered elevated at values >150 mg/dL. The individuals with a glucose level ≥126 mg/dL or those under regular use of insulin and/or oral hypoglycemic agents were classified as diabetic. Fasting glucose levels between 110 and 125 mg/dL were considered indicative of impaired glucose tolerance [19].

Participants underwent conventional electrocardiogram recording at rest using a computerized device (Schiller® AT-10 EKG, Baar, Switzerland) with 12 leads, which allowed for the automatic retrieval of several measurements. The Sokolow-Lyon- Rappaport index (SV1 or SV2 + RV5 or RV6) was used to classify individuals as having left ventricular hypertrophy (LVH), which was considered to be present when the index was >3.5 mV [20].

For continuous variables, the mean difference between genders was analyzed using the Student's t-test for independent samples. The chi-squared (X^2^) test was used for dichotomous variables (smoking, diabetes, and LVH) and the comparison of the proportions of the main cardiovascular risk factors among the groups. A one-way analysis of variance (ANOVA) was used to determine differences between three or more categorized groups. The analysis of differences within each ANOVA was conducted using Tukey’s post hoc test. An analysis of covariance (ANCOVA) was used to assess the effects of age and gender on BMI, SBP, and DBP in the different socioeconomic groups, as well as the effects of SBP and gender on the Sokolow-Lyon indices of the groups. The significance level for all tests was set at alpha <0.05. Statistical analyses were performed using SPSS for Windows version 16.0.

## Results

Table 1 lists the demographic characteristics of the 615 individuals (294 men and 321 women) included in the study, age ranging from 20–72 years (age mean = 44.5 ± 10.6 years, without significance difference between genders; P = 0.176). Individuals with a low educational level accounted for 34.6% of the sample population, those with a medium educational level constituted 24.4%, and those with a high educational level constituted 41.0%. Education levels were similarly distributed by genders (X^2^ = 1.93, P = 0.926).

**Table 1:** Demographic, anthropometric, and biochemical characteristics of the participants according to gender

	**Men**	**Women**	***P***	**All**
**N (%)**	294 (47.8)	321 (52.2)	0.392	615 (100)
**Age (years)**	45.1±11.1	44.0±10.1	0.176	44.5±10.6
**Educational level**			0.926	
Low	110 (37.4)	103 (32.1)		213 (34.6)
Medium	69 (23.5)	81 (25.2)		150 (24.4)
High	115 (39.1)	137 (42.7)		252 (41.0)
**Socioeconomic class**			0.392	
Low	81 (27.6)	73 (22.7)		154 (25.0)
Middle	77 (26.2)	79 (24.6)		156 (25.4)
Upper middle	66 (22.4)	86 (26.8)		152 (24.7)
Upper	70 (23.8)	83 (25.9)		153 (24.9)
**WC (cm)**	80.1±12.9	83.9±13.5	<0.001	82.1±13.3
**HC (cm)**	91.5±9.4	99.5±11.4	<0.001	95.7±11.3
**BMI (kg/m**^**2**^**)**	24.1±4.3	27.1±5.8	<0.001	25.7±5.4
**Glucose (mg/dL)**	94.9±20.0	93.2±21.8	0.313	94.0±21.0
**TC (mg/dL)**	189.5±41.4	193.2±36.5	0.240	191.5±38.9
**HDL-c (mg/dL)**	44.2±10.3	47.6±11.2	<0.001	46.0±10.9
**LDL-c (mg/dL)**	125.0±41.8	125.9±38.7	0.796	125.5±40.2
**TG (mg/dL)**	101.7±41.7	98.7±38.4	0.340	100.2±40.0

*WC* waist circumference, *HC* hip circumference, *BMI* body mass index, *TC* total cholesterol, *HDL*-*c* high density lipoprotein cholesterol, *LDL-c* low-density lipoprotein cholesterol, *TG* triglycerides, Proportions were expressed as number of individuals (percentage) and analyzed using the chi-square test. Continuous variables are reported as mean ± standard deviation, and analyzed using the Student’s t-test for independent samples.

Mean values of WC (80.1 ± 12.9 vs. 83.9 ± 13.5 cm), HC (91.5 ± 9.4 vs. 99.5 ± 11.4 cm), and BMI (24.1 ± 4.3 vs. 27.1 ± 5.8 kg/m^2^) were higher in the women (P < 0.001).

With regard to WC, 39.8% of the sample showed values above the recommended level, and women had a significantly higher (P < 0.05) proportion of high WC (23.4%) and markedly high WC (38.9%) compared with the values found in the men (8.8% vs. 6.5%). Regarding the biochemical data for the sample group, gender differences were found with regard to HDL level; namely, the women had a higher average value than the men (47.6 ± 11.2 vs. 44.2 ± 10.3 mg/dL; P < 0.001). The mean blood glucose levels, total cholesterol, LDL, and TG were similar in both genders.

Table 2 shows the anthropometric, clinical, and biochemical characteristics according to the socioeconomic level. Age was different (P < 0.001) among individuals of different socioeconomic groups. The three anthropometric indexes related to body weight increased (P<0.001) from the lowest to the upper socioeconomic group while blood pressure and the SKI showed an opposite tendency when only the three lowest classes were considered, even after adjustment for age and gender. In addition there were still differences in SBP and DBP after adjustment for age, gender, BMI, as well as in the SLI after adjustment for SBP, age, and gender. Biochemical variables, tended to remain stable along socioeconomic levels, except for glucose levels which were significantly higher in the upper class.

**Table 2:** Anthropometric, clinical, and biochemical characteristics according to the socioeconomic class

**Class**	**Low**	**Middle**	**Upper middle**	**Upper**	***P***
**N (%)**	154 (25.0)	156 (25.4)	152 (24.7)	153 (24.9)	0.967
**Age (years)**	48.4 ± 9.7	43.4 ± 10.6	40.3 ± 10.6	45.9 ± 9.7	< 0.001
**WC (cm)**	77.6 ± 12.7	80.8 ± 13.2	82.9 ± 13.4	87.1 ± 12.2	< 0.001
**HC (cm)**	90.5 ± 11.0	94.5 ± 10.9	97.2 ± 10.5	100.7 ± 10.2	< 0.001
**BMI (kg/m**^**2**^**)**	23.9 ± 5.0	25.2 ± 5.4	26.1 ± 5.3	27.6 ± 5.0	< 0.001
**SBP (mmHg)**	141.7 ± 27.7	136.6 ± 26.7	128.7 ± 22.7	131.7 ± 19.6	< 0.001
**DBP (mmHg)**	85.0 ± 14.3	83.5 ± 14.9	80.1 ± 13.8	81.8 ± 12.4	< 0.05
**SKI (mm)**	30.0 ± 9.6	28.0 ± 9.1	24.9 ± 8.1	25.0 ± 8.2	< 0.001
**Glucose (mg/dL)**	93.4 ± 15.3	93.2 ± 23.3	91.3 ± 15.8	98.1 ± 26.8	< 0.05
**TC (mg/dL)**	194.4 ± 39.5	186.1 ± 37.4	193.0 ± 39.6	192.4 ± 38.9	0.242
**HDL-c (mg/dL)**	46.4 ± 11.4	46.4 ± 10.7	47.3 ± 11.0	43.8 ± 10.4	< 0.05
**LDL-c (mg/dL)**	127.7 ± 41.4	120.1 ± 38.5	126.1 ± 40.9	128.0 ± 39.7	0.273
**TG (mg/dL)**	101.4 ± 34.9	98.0 ± 38.3	98.1 ± 42.1	103.2±44.2	0.588

*N* number of individuals, *WC* waist circumference, *HC* hip circumference, *BMI* body mass index, *SBP* systolic blood pressure, *DBP* diastolic blood pressure, *SKI* Sokolow-Lyon index, *TC* total cholesterol, *TG* triglycerides. Data are reported as the mean ± standard deviation.

The prevalence of cardiovascular risk factors according to gender is shown in Table 3.

**Table 3:** Prevalence of cardiovascular risk factors according to gender

**Risk factors**	**Men (N = 294)**	**Women (N = 321)**	**All (N = 615)**
	**N (%)**	**N (%)**	**N (%)**
**Hypertension**	136 (46.3)	142 (44.2)	278 (45.2)
**TC ≥ 240 mg/Dl**	31 (10.5)	37 (11.5)	68 (11.1)
**LDL-c ≥ 160 mg/dL**	61 (20.8)	60 (18.9)	121 (19.8)
**Low HDL-c (mg/dL)**	108 (36.9)*	200 (62.3)	308 (50.1)
**TG ≥ 150 mg/dL**	37 (12.6)	28 (8.7)	65 (10.6)
**Smoking history**	30 (10.2)*	14 (4.4)	44 (7.2)
**Diabetes**	16 (5.5)	19 (5.9)	35 (5.7)
**Overweight**	80 (27.3)	100 (31.2)	180 (29.3)
**Obesity**	27 (9.2)*	93 (29.0)	120 (19.6)
**Sedentary lifestyle**	244 (83.0)*	292 (91.0)	536 (87.2)
**LVH**	94 (32.0)*	29 (9.0)	123 (20.0)

Hypertension: SBP ≥140 mmHg and/or DBP ≥90 mmHg or normotensive on antihypertensive medication. TC: total cholesterol; LDL-c: low-density lipoprotein cholesterol; HDL-c: low high-density lipoprotein cholesterol (men, <40 mg/dL; women, <50 mg/dL); TG: triglycerides; diabetes: fasting glucose ≥126 mg/dL; overweight: BMI between 25 and 29.9 kg/m^2^; obesity: BMI ≥ 30.0 kg/m^2^; LVH: left ventricular hypertrophy (Sokolow-Lyon index ≥3.5 mV). (*)P<0.01 men vs women.

The overall prevalence of hypertension was 45.2% (n = 278), with similar proportion in men (46.3%, n = 136) and women (44.2%, n = 142). Higher prevalence of hypertension was found in the lowest socioeconomic classes (Table 4). The overall prevalence of hypercholesterolemia was 11.1%, and the proportions were similar in both genders (men, 10.5%; women, 11.5%) and unrelated to socioeconomic classes.

**Table 4:** Prevalence of cardiovascular risk factors according to socioeconomic class

**Socioeconomic class**	**Low**	**Middle**	**Upper middle**	**Upper**	***P***
Risk factor	N (%)	N (%)	N (%)	N (%)	
Hypertension	86 (30.9)	68 (24.5)	55 (19.8)	69 (24.8)	< 0.05
TC ≥ 240 mg/dL	20 (13.0)	11 (07.1)	19 (12.5)	18 (11.8)	0.483
LDL-c ≥ 160 mg/dL	34 (22.2)	26 (17.0)	29 (19.1)	32 (20.9)	0.899
Low HDL-c (mg/dL)	72 (23.4)	77 (25.0)	70 (22.7)	89 (28.9)	0.128
TG ≥ 150 mg/dL	16 (24.6)	16 (24.6)	13 (20.0)	20 (30.8)	0.641
Smoking history	22 (50.0)	10 (22.7)	08 (18.2)	04 (09.1)	< 0.001
Diabetes	05 (14.3)	06 (17.1)	11 (31.4)	13 (37.1)	0.134
Overweight	33 (18.3)	42 (23.3)	43 (24.0)	62 (34.4)	< 0.001
Obesity	18 (15.0)	26 (21.7)	34 (28.3)	42 (35.0)	< 0.001
Sedentary lifestyle	147 (95.5)	139 (89.1)	132 (86.8)	118 (77.1)	< 0.001
LVH	47 (38.2)	32 (26.0)	22 (17.9)	22 (17.9)	< 0.001

Hypertension: SBP ≥140 mmHg and/or DBP ≥90 mmHg or normotensive on antihypertensive medication; TC: total cholesterol; LDL-c: low-density lipoprotein cholesterol; HDL-c, high-density lipoprotein cholesterol (men, <40 mg/dL; women, <50 mg/dL); TG: triglycerides; diabetes: fasting glucose ≥126 mg/dL; overweight: BMI between 25 and 29.9 kg/m^2^; obesity: BMI ≥ 30.0 kg/m^2^; LVH, left ventricular hypertrophy (Sokolow-Lyon index ≥ 3.5 mV).

The prevalence of high LDL levels did not differ between genders (men, 20.8%; women, 18.9%) or between socioeconomic classes. However, 24.8% of the participants had borderline values (men, 22.4%; women, 26.9%). There was an overall low HDL fraction prevalence of 50.1%. This proportion was higher (X^2^ = 3.840, P < 0.05) in women (62.3%) than men (36.9%). No significant differences were found between the socioeconomic classes regarding the prevalence of low HDL (low 46.8%, middle 49.4%, upper middle 46.1%, upper 58.2%; P = 0.128).

Hypertriglyceridemia was prevalent in approximately 10.6% of the entire sample, and the prevalence were similar between genders (X^2^ = 2.422, P = 0.658) and among the four socioeconomic classes.

Active smoking was related by 7.2% of the sample (Table 3), with a higher proportion in men (men = 10.2% vs women = 4.4%; X^2^ = 4.430, P<0.05). Prevalence of smoking decreased significantly from the lowest socioeconomic class to the upper one (14.3% to 2.6%; P<0.001, Table 4). Approximately 20.3% (n = 125) of the sample group were former smokers, and more men (X^2^ = 4;43; P<0.001) were former smokers (28.6%, n = 84) than women (12.8%, n = 41).

In the sample of 615 participants, 88.3% (n = 543) showed normal blood glucose levels, 6.0% (n = 37) had decreased glucose tolerance, 3.3% (n = 20) were diabetics under treatment, and 2.4% (n = 15) were diabetics who were unaware of their status until the time of this evaluation. Thus, the overall prevalence of diabetes mellitus was 5.7%, and the distribution of diabetes between the genders was similar (men 5.5%, women 5.9%). Among the socioeconomic classes (low 3.2%, middle 3.8%, upper middle 7.2%, upper 8.5%; P = 0.134), although no significant differences were found, it is noteworthy that the upper socioeconomic class had a relatively higher prevalence of diabetes than the low socioeconomic class.

Overweight was found in 29.3% of the sample population, with similar distribution in both genders (X^2^ = 1.151, P = 0.886). Obesity, however, was three times more prevalent in women (Table 3). A clear increase of prevalence of overweight and obesity was observed from the lowest to upper socioeconomic classes (Table 4), where 68% showed excessive body weight (body mass index ≥25 kg/m^2^).

The overall prevalence of sedentary lifestyle was 87.2%, and it was higher (X^2^ = 8.712, P< 0.05) in women (91.0%;) than in men (83.0%). Individuals of the low socioeconomic class were more inactive than those in the upper socioeconomic class. The overall prevalence of LVH was 20.0%, and it was more prevalent (X^2^ = 50.46; P<0.05) in men (32.0%) than in women (9.0%). There was a higher percentage of LVH in the low socioeconomic class (30.5%) than in the upper socioeconomic class (14.4%).

**Figure 1:**
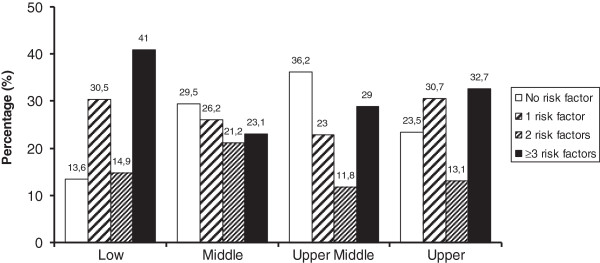
Proportion of cardiovascular risk factors according to socioeconomic classes.

Distribution of the number of cardiovascular risk factors is shown in Table 5, with similar values between genders. It is important to note that only 25.7% of the participants were free from modifiable cardiovascular risk factors and around 50% of the sample showed two or more risk factors to cardiovascular disease. Figure 1 illustrates the proportion of cardiovascular risk factors according to socioeconomic classes. We observed that the presence of three or more risk factors was higher in the low socioeconomic class (X^2^ = 23.766, P< 0.05) than in the upper class.

**Table 5:** Cardiovascular risk factors according to gender

**Risk factors**	**Men**	**Women**	**All**
	N (%)	N (%)	N (%)
None	74 (25.2)	84 (26.2)	158 (25.7)
One risk factor	78 (26.5)	92 (28.5)	170 (27.7)
Multiple risk factors			
Two	49 (16.6)	45 (14.0)	94 (15.2)
Three	57 (19.4)	62 (19.3)	119 (19.4)
≥ Four	36 (12.3)	38 (12.0)	74 (12.0)
Total	294 (100)	321 (100)	615 (100)

No difference between genders was found (chi-square test).

## Discussion

Determination of the prevalence of cardiovascular risk factors in adults has been conducted in several parts of the world; however, few studies were performed in African populations and to our knowledge, there have been no studies conducted in Angola. In this study, the prevalence of cardiovascular risk factors was investigated in the adult population of public-sector workers at UAN, which is the oldest public institution of higher education in Angola.

Illiteracy and low education levels are prevalent in low-income countries, and it is known that there is a strong correlation of illness and death with poverty. A low level of education is one of the major determinants of disease and mortality because it is associated with malnutrition and acute and chronic infections [21]. Although the participants of this study worked in an institution of higher education, the prevalence of low education level was 34.6%. This figure is similar to the Angolan norm because, according to the Inquiry into the Welfare of the Population [10], illiteracy in the Angolan population aged 15 years and older was 34.4%. In Africa, the illiteracy rate varies from 10 to 87.2% [22]. In a study conducted in India, the illiteracy rate was found to be 45.3% for women and 17.0% for men, and there was an inverse relationship between education and mortality from all causes [23]. Education increases people’s communication skills, reduces inequalities in the knowledge of disease transmission, and provides new opportunities in the production and marketing sectors. The improvement of education is thus crucial for the prevention, early diagnosis, and adequate management of chronic disease risk factors.

Therefore, education serves as a social catalyst.

Socioeconomic inequalities remain one of the main constraints to sustainable development in Africa. The percentage of our sample group in the low socioeconomic class was slightly less than that reported by IBEP [10], where the proportion of the Angolan population living below the national poverty line was found to be 33.6%.

Previous studies have reported that more individuals from low socioeconomic groups die from acute coronary events compared with individuals from high socioeconomic groups [24]. Angola is a low-income country, where many regions remain at an early stage of epidemiological transition, with sequelae from a long civil war and large pockets of poverty. These factors certainly negatively affect Angola’s sustainable development, particularly taking into account the early death of the work force.

The prevalence of hypertension in the current study was 45.2%, which is a higher value than those reported in other African countries, such as South Africa [25], Uganda [26], Nigeria [27], Ethiopia [28], and Ghana [29], where the prevalence of hypertension varied from 13.7% in rural areas to 30.5% in urban areas. The SBP of individuals in the low socioeconomic class was higher than that of individuals in the upper class. Similarly, the prevalence of hypertension was higher in the low socioeconomic class than in the upper class. These findings may be associated with psychosocial factors related to poverty, chronic stress at work in activities that involve physical exhaustion [30], and limited access to antihypertensive drugs.

The mean blood glucose level in the upper class was higher than those in the low class. The prevalence of diabetes in our study was similar in both genders. These values are higher than those found in a rural community of Angola (2.8%) [31]; however, our values are close to those found in the adult population of urban Ghana (6.3%) [32]. Nevertheless, the prevalence of diabetes in the present study was lower than in Cameroon (10.4%) [33] and Afro-Surinamese (14.2%) [34].

The benefits of low cholesterol in the primary and secondary prevention of CVD are clear. The mean cholesterol level found in this study was normal according to the previously defined points. The overall prevalence of hypercholesterolemia was lower than the values previously reported for Nigeria (28.3%) [27] and African-Americans (28.9%) [35]. Although no significant difference was found, the prevalence of hypercholesterolemia in the low socioeconomic class was slightly higher than that in the upper socioeconomic class. The prevalence of low HDL was high, and more women were affected than men. This discrepancy is likely because the majority of women included in the study were premenopausal phase since the mean age of the women was 44.0 ± 10.1 years. Regarding socioeconomic status, although no significant difference was found, there was a trend to higher HDL levels in the upper class. The proportion of high LDL (19.8%) was similar between genders and between different socioeconomic classes.

The prevalence of hypertriglyceridemia was similar to the prevalence found in the Nigerian population (15.0%) [27] but lower than the prevalence found in the third National Survey on Health and Nutrition (NHANES III) in African-American men and women (21.0 and 14.0%, respectively) [35].

The BMI was higher in women than in men, and a direct relationship with socioeconomic status was found, namely, BMI progressively increased as socioeconomic status increased. This finding was marked in men and was also observed with regard to waist circumference (WC), where 62.3% of women and 15.3% of men exhibited values higher than recommended [16]. Angola is a low- income country, but overweight and obesity appear to coexist with undernutrition and malnutrition. In 2006, the overall prevalence of malnutrition was 44.0% [36], and 20.0% of children die from this cause [37]. Excessive weight increases the probability of obesity and weight-related disorders. In this study, the prevalence of obesity was higher in women than in men.

Overweight and obesity are prevalent in many countries, and according to Nishida and Mucavele [38], the prevalence of obesity is higher in women than in men in countries such as Egypt (33.0 vs. 12.6%), South Africa (30.1 vs. 9.4%), and the Seychelles Islands (28.2 vs. 8.5%). Nutrition plays a significant role in many risk factors associated with CVD. Considering that approximately 49.0% of the sample population had body weights above the recommended values, it is necessary to take preventative measures to mitigate future complications. Physical inactivity is a malady of the modern world because the comfort provided by various types of technology and the consumption of highly caloric industrialized food contribute to weight gain and, consequently, a decreased quality of life. The prevalence of physical inactivity found in this study was very high, and women were more inactive than men. Similar data were reported for the population of Porto, Portugal (84.0%) [39].

The prevalence of smoking was low, although it was higher in men than in women. These data are similar to those reported by Tran et al. in Ethiopia [28] and by Addo et al. in Ghana [40]. In general, smoking prevalence among African women is low, likely because smoking is not a culturally well-accepted habit for this gender. Nevertheless, it is necessary to continue policies aimed at reducing the use of tobacco even further.

The progressive remodeling of the left ventricle (LV) is directly related to the further deterioration of cardiac performance and a less favorable outcome in presence of CVD [41]. LVH is an adaptive response of the myocardium to increased cardiac work, resulting in increased cardiac mass, which can lead to ventricular arrhythmias, myocardial ischemia, systolic and diastolic ventricular dysfunction, and sudden death [42]. The prevalence of LVH was higher in men than in women, and the low socioeconomic class was more affected than the upper class. The high prevalence of hypertension found in the low socioeconomic class is due to discrepancies in the prevalence of LVH among socioeconomic classes.

This study demonstrated the existence of a high prevalence of modifiable risk factors for cardiovascular disease among the study participants, such as hypertension, low HDL level, overweight, obesity, sedentary lifestyle, and LVH, rendering them more likely to be affected by a cardiovascular event, especially when associated with low socioeconomic status, as the majority of individuals with this status had three or more risk factors. Because this sample is a convenience sample, generalization of the findings in the present study to a wider Angolan population may be limited; however, our findings serve as indicators of the health of workers in Angola because all socioeconomic groups in this country were represented.

## Conclusions

Our study suggests the existence of a high prevalence of multiple risk factors for cardiovascular disease in apparently healthy public-sector workers in Angola, and that the socioeconomic groups with lower incomes were more affected by hypertension, smoking, and LVH. The coexistence of multiple risk factors in this population indicates the necessity to intensify efforts for the prevention, early identification, and control of risk factors, especially in the lower-income segments of the population, which represents the priority group for intervention and the promotion of cardiovascular health.

## Competing interests

The authors declare that they have no competing interests in this work.

## Authors’ contributions

DPC, PM, and JGM designed the study. DPC and AVLF explained the study to the workers at the Schools and Institutes. ABTS, MAAG, CCFS, AVLF, and PM participated in the data collection and managed the equipment in the work space. DPC wrote the article and was responsible for data management, interpretation of the results, and conducting the literature review. MPB and SLR assisted with the data analysis and the writing of the paper. JGM coordinated the study and participated in all stages of the writing. All authors read and approved the final manuscript.

## Pre-publication history

The pre-publication history for this paper can be accessed here:

http://www.biomedcentral.com/1471-2458/13/732/prepub
